# Genetic Variants in the MTHFR and WNK1 Genes and Their Contribution to Hypertension Susceptibility

**DOI:** 10.7150/ijms.133244

**Published:** 2026-06-17

**Authors:** Laith N. AL-Eitan, Ola M. Al-Sanabra, Fouad A. Almomani, Maryam K. Alasmar, Mariam R. Alrejjal, Zeina F. Obeid, Ra'ed I. AlRabadi

**Affiliations:** 1 Department of Biotechnology and Genetic Engineering, Jordan University of Science and Technology, Irbid 22110, Jordan.; 2 Department of Medical Laboratory Sciences, Faculty of Allied Medical Sciences, Al-Balqa Applied University, Al-Salt 19117, Jordan.

**Keywords:** Hypertension, MTHFR, polymorphism, WNK1.

## Abstract

**Background:**

Hypertension (HTN) is a common and complex disorder influenced by multiple genetic and environmental factor, where the underlying mechanisms of its etiology remain incompletely understood although the identification of several contributing elements. This research sought to evaluate the possible relationship between genetic polymorphisms in methylenetetrahydrofolate reductase (MTHFR) and With-No-Lysine Kinase 1 (WNK1) genes and susceptibility to hypertension.

**Method:**

Genomic DNA was extracted from blood samples collected from 220 individuals with hypertension and 220 normotensive controls. Genotyping of MTHFR (rs1801133 and rs1801131) and WNK1 (AluYb8) polymorphisms was performed using direct PCR and PCR-RFLP techniques. The resulting data were subjected to appropriate statistical analyses.

**Results:**

A statistically significant association was identified between the rs1801131 polymorphism of the MTHFR gene and susceptibility to hypertension (p = 0.0006). This association remained significant under the codominant, dominant, and recessive genetic models, with all p-values < 0.016. Furthermore, the CC haplotype of the MTHFR gene showed a significant association with hypertension (OR = 2.02, p = 1e-04).

**Conclusion:**

These findings indicate that the MTHFR rs1801131 polymorphism is significantly associated with hypertension susceptibility and may represent a potential genetic marker. This highlights its relevance for future studies exploring genotype-driven risk assessment and personalized approaches to hypertension management.

## Introduction

Hypertension (HTN) represents a chronic and multifactorial ailment arising from a confluence of environmental and genetic factors. It affects over a quarter of the adult population, increasing the risk of consequential maladies such as stroke, renal disease, coronary heart disease and heart failure 1. The principal determinants of blood pressure are cardiac output and total peripheral resistance, with diagnostic thresholds generally set at diastolic blood pressure (DBP) ≥ 90 mmHg and systolic blood pressure (SBP) ≥ 140 mmHg 2, 3. It is worth noting that over 90% of hypertension instances fall under the classification of essential hypertension, or hypertension with no recognized etiology 4.

Due to population aging and increased exposure to lifestyle risk factors such as poor diets, the prevalence of hypertension is increasing globally 5. Essential hypertension is estimated to affect approximately 25-35% of the adult population and 60-70% of individuals beyond the seventh decade of life across both developed and developing countries 6. However, not every country is experiencing the same changes in the prevalence of hypertension 7.

Several sources provide evidence for the genetic impact on blood pressure. Despite the fact that different genes and genetic variables have been associated with the emergence of essential hypertension, a particular person's disease is most likely the result of numerous genes working together 8. Hypertension is estimated to have a heritable component of up to 60%. Identifying genetic variations associated with blood pressure regulation is important for two main reasons: first, to recognize individuals at high genetic risk for early diagnosis, prevention and treatment; and second, to elucidate the molecular mechanisms underlying hypertension, which may reveal potential targets for prevention, therapy and diagnostics 9.

The MTHFR (methylenetetrahydrofolate reductase) gene is located on the distal region of the short arm of chromosome 1 (1p36.3). It encodes an important enzyme implicated in folate and methionine metabolism. The gene spans around 20.374 kb and involves 12 exons. Its mRNA transcript is about 7,150 base pairs in length, which encodes a protein of 656 amino acids 10. It is intricately involved in the metabolic pathways of both homocysteine (Hcy) and folate. MTHFR acts as a key enzyme that influences the metabolism of homocysteine (Hcy) 11. The concentration of serum homocysteine has been established as correlating with the incidence of hypertension 12. Homocysteine, a sulfur-containing amino acid, has the potential to cause damage to blood vessels 13. Elevated levels of homocysteine have a synergistic effect with hypertension, notably increasing the risk of vascular diseases 14, 15.

The maintenance of homeostasis surrounding the vascular endothelium depends on the balance between the availability of nitric oxide (NO) and the presence of oxidizing reactive oxygen species (ROS). Nitric oxide contributes to vasodilation and mitigates platelet aggregation and adhesion within the vascular endothelium and plays an important role in hypertension. Circulating levels of NO are influenced by the concentrations of Hcy and MTHFR 16.

The MTHFR enzyme is produced by the MTHFR gene, and its function is significantly influenced by variations in this gene. Specifically, two widely studied SNPs in the MTHFR gene known as C677T (rs1801133) and A1298C (rs1801131) have been found to be associated with decreased enzyme activity and increased plasma homocysteine levels 17, 18. The MTHFR gene rs1801133 variant is located in exon 4 and involves a cytosine-to-thymine substitution, resulting in an alanine-to-valine change at codon 222 (Ala222Val). The resulting protein is thermally unstable with low enzyme activity and hence less MTHFR function and increased concentration of homocysteine (19, 20). Similarly, the rs1801131 polymorphism occurs in exon 7 and is characterized by an adenine-to-cytosine substitution at nucleotide position 1298, causing a glutamate-to-alanine substitution at codon 429 (Glu429Ala) within the C-terminal regulatory domain. This variant is also related to decreased enzymatic activity, even though to a lesser degree than rs1801133 (20, 21).

The human WNK1 (With-No-lysine Kinase 1) gene located on chromosome 12p13 and consisting of 29 exons covers approximately 160 kb. It encodes multiple transcripts that originate from different promoters 22-24. WNK1 is a serine/threonine protein kinase that plays a role in regulating sodium and potassium transport in the distal convoluted tubules and cortical collecting ducts of kidney nephrons. This function contributes to the modulation of blood pressure 23, 25. It is highly expressed in many different tissues, with particularly high concentrations being found in the kidney and the cardiovascular system. The expression of WNK1 is localized mainly to the distal nephron of the kidney, particularly within the region involved in NaCl reabsorption. Research indicates that elevated WNK1 expression could potentially enhance NaCl reabsorption, consequently promoting volume expansion-induced hypertension 23.

There are some common polymorphisms and haplotypes of WNK1 gene that are associated with blood pressure and the severity of hypertension. Among the polymorphisms of the WNK1 gene, the insertion of the AluYb8 element in the WNK1 intron 10 was observed to affect the variability of blood pressure of individuals 26.

Despite growing evidence of genetic contributions to hypertension, studies exploring relevant gene variants in Middle Eastern populations remain scarce. We hypothesize that MTHFR (rs1801133 and rs1801131) and WNK1 (AluYb8) polymorphisms may influence susceptibility to hypertension in the Jordanian population.

## Methods

### Study Subjects

A total of 220 patients diagnosed with hypertension and 220 normotensive controls were enrolled from the Jordanian Arab population. Participants were recruited during routine clinical visits at the Cardiac Clinic and Coronary Care Department of King Abdullah University Hospital (KAUH) in Irbid, Jordan. All patients included in the study were diagnosed with hypertension and were undergoing treatment with various classes of antihypertensive medications for a minimum duration of one year; however, based on the class of antihypertensive medication prescribed (ACEIs, ARBs, CCBs, BBs and TDs) participants were categorized into five groups according to American Heart Association (AHA) and Joint National Committee (JNC) guidelines implemented at KAUH.

A total of 220 unrelated Jordanian patients were enrolled in this study after excluding individuals who did not meet the inclusion criteria. The inclusion criteria specified having a confirmed clinical diagnosis of hypertension, available medical data recorded in the KAUH registry system, and being 35 years old or above. Exclusion criteria specified participants who did not consent to the study, had missing data, had relatives within the second degree of kinship, or did not adhere to prescribed anti-hypertension treatment. Additionally, a control group comprising 220 unrelated, normotensive adult volunteers was recruited from the general population. Inclusion criteria required that participants be adults without a prior diagnosis of hypertension, cardiovascular disease, or chronic kidney disease. Individuals receiving antihypertensive medications or with a history of secondary hypertension were excluded. Participants who refused to sign the informed consent form or were not of Jordanian nationality were also excluded. Approval for the study design and proposal was granted by the Human Ethics Committee of Jordan University of Science and Technology, Irbid, Jordan. All individuals enrolled in the study gave their written informed consent.

### Sample Preparation and Genotyping

Genomic DNA extraction was carried out from frozen whole blood samples, following the manufacturer's instructions for the Puregene® Blood Core Kit A (Qiagen, USA). DNA concentration and purity were evaluated using agarose gel electrophoresis and the NanoDrop ND-1000 spectrophotometer (BioDrop, UK).

Genotyping of the MTHFR (rs1801133 and rs1801131) and WNK1 (AluYb8) polymorphisms was conducted using direct PCR and PCR-restriction fragment length polymorphism (PCR-RFLP) methods. Each PCR reaction for all examined polymorphisms was carried out in a final volume of 25 µL, containing 6.5 µL nuclease-free water, 12.5 µL of 2× PCR Master Mix (Promega, Madison, WI, USA), 1.5 µL of each forward and reverse primer (10 µM stock concentration), and 3 µL of genomic DNA (~60 ng). Primer sequences for the analyzed polymorphisms are listed in Table 1.

Reactions were performed in a thermal cycler (Applied Biosystems Veriti, Thermo Fisher Scientific, Waltham, MA, USA) under the following conditions: initial denaturation at 95°C for 10 min, followed by 35 cycles of 95°C for 30 s (denaturation), 30 s of annealing at 60°C for MTHFR (rs1801133 and rs1801131) and 58.8°C for WNK1, and 72°C for 30 s (extension), with a final elongation at 72°C for 5 min. PCR products of rs1801133 and rs1801131 (10 µL) were digested with the appropriate restriction enzyme (HinfI for rs1801133 and MboII for rs1801131; Thermo Fisher Scientific, Waltham, MA, USA) in a 10 µL reaction mixture at 37°C for 2 h. The digested products, along with WNK1 PCR products, were resolved on a 3% agarose gel (HiMedia, Mumbai, India), and the resulting fragment sizes are shown in Table 1. Genotyping quality was assessed by re-analyzing a randomly selected subset of 20% of samples, which yielded 100% concordance between runs. Samples with unclear or ambiguous results were re-genotyped to ensure accurate genotype assignment. The overall call rate for all polymorphisms was 100%.

### Statistical Analysis

Various statistical tests were employed, and different software applications were used for these analyses. SNPStats, a web-based statistical tool accessible at http://www.snpstats.net/start.htm, was used to test for Hardy-Weinberg Equilibrium, estimate allelic and genotypic frequencies, test genetic models, and study the association of haplotypes with disease predisposition. The estimates of association were described as Odds Ratios (ORs), with 95% Confidence Intervals (CIs). The correlations of genotypes with phenotypes were analyzed statistically by using Pearson chi-square tests, together with a one-way ANOVA test, via SPSS software version 26.0 (SPSS, Inc., Chicago, IL). A multivariable binary logistic regression model was applied to evaluate the independent effect of the studied polymorphisms on disease risk while adjusting for potential confounding variables. For multiple comparisons, the effective number of polymorphisms was estimated according to a previously described method 27. For correcting the significance level, the Bonferroni test was applied, whereby the significance level was calculated as α/n, where α = 0.05 and n corresponds to the total number of performed tests 28.

## Results

### Population Characteristics and Hardy-Weinberg Test

The control group consisted of 220 healthy individuals (mean age: 58.75 ± 10.28 years), of whom 37.3% were male. The hypertensive group included 220 patients (mean age: 58.84 ± 10.39 years), with 58.2% male participants. Body mass index (BMI) was assessed for both groups, with mean values of 25.04 ± 4.28 for the control group and 31.60 ± 6.04 for the hypertensive patients. Baseline characteristics of the participants are detailed in Table 2.

Hardy-Weinberg equilibrium (HWE) analysis was conducted for all investigated SNPs, and no significant deviations were observed in either the case or control groups (p > 0.05). Consequently, all SNPs were included in subsequent analyses, as presented in Table 3.

### Genetic Association of WNK1 and MTHFR Polymorphisms with Hypertension

The distributions of genotypes and allele frequencies for MTHFR and WNK1 gene polymorphisms are presented in Table 4. A significant association was identified for the MTHFR rs1801131 polymorphism. The A allele was significantly more frequent in controls compared to cases (80% vs. 68%), while the C allele was more prevalent among patients (32% vs. 20%) (p = 0.0001). Genotype frequencies also showed a significant difference between the two groups (p = 0.0006), with the AA genotype being more common in the control group while the CC genotype was more frequent in the case group. These findings suggest that the CC genotype may be associated with increased susceptibility to hypertension.

In contrast, for the MTHFR rs1801133 polymorphism, no statistically significant differences were observed between hypertension cases and controls at either the allele level (p = 0.776) or genotype level (p = 0.848). Regarding the WNK1 (AluYb8) polymorphism, no statistically significant differences were observed between cases and controls at either the allele level (p = 0.074) or genotype level (p = 0.173), suggesting no association with hypertension risk in this study population.

### Genetic Model Analysis

Genetic models were employed to assess the association between MTHFR and WNK1 polymorphisms and hypertension risk. Odds ratios estimating the risk of hypertension for each MTHFR and WNK1 polymorphism are provided in Table 5. Significant associations were identified between MTHFR rs1801131 and hypertension under the codominant (OR = 1.69 and 3.70; p = 5e-04), dominant (OR = 1.92; p = 8e-04), and recessive (OR = 3.01; p = 0.0036) genetic models, indicating notable differences between cases and controls, with the CC genotype appearing to be associated with an increased risk of hypertension. The remaining polymorphisms did not demonstrate significant associations with hypertension under any of the genetic inheritance models.

### Haplotype Analysis of MTHFR Gene

Haplotype structures of the rs1801133 and rs1801131 SNPs in the MTHFR gene were analyzed to assess their potential contribution to hypertension susceptibility. A significant association was identified between the CC haplotype and increased susceptibility to hypertension (OR = 2.02; p = 1e-04). However, no significant associations were found for the remaining haplotype structures, as presented in Table 6.

### Association of WNK1 and MTHFR Polymorphisms with Hypertension-Related Clinical Outcomes

The analysis aimed to evaluate whether different genotypes of the studied polymorphisms are associated with variability in clinical and anthropometric characteristics within the hypertension patient group. Table 7 presents the associations between specific polymorphism genotypes and clinical outcomes. For the WNK1 polymorphism (AluYb8), significant correlations were observed with pulse rate (p = 0.0147), HDL cholesterol (p = 0.0080) and HbA1c (p = 0.0103). In contrast, no significant associations were observed between the MTHFR polymorphisms (rs1801133 and rs1801131) and any hypertension-related clinical outcomes. These results indicate that particular genetic polymorphisms in the WNK1 gene could affect some clinical trait associated with hypertension, thus underscoring the possible involvement of genetic factors in its pathophysiology.

### Multivariable Logistic Regression Analysis of Predictors for Hypertension Risk

Multivariable binary logistic regression analysis was performed to evaluate the independent effects of genetic polymorphisms and covariates on hypertension risk. The model included age, BMI, gender, smoking status and genotypes of MTHFR (rs1801131, rs1801133) and WNK1 (AluYb8), as presented in Table 8.

Across all models, BMI and gender remained significantly associated with hypertension risk. Increased BMI was consistently associated with higher odds of hypertension (OR = 1.29-1.30, p < 0.001), while males had significantly higher odds compared to females (OR = 3.19-3.60, p < 0.001). In contrast, age and smoking were not significantly associated with (p > 0.0167).

Regarding genetic polymorphisms, no significant correlation was observed between any of the studied SNPs and hypertension risk after Bonferroni correction. Specifically, for MTHFR (rs1801131), neither the C/C nor A/C genotypes showed a significant association compared to the A/A reference genotype (p > 0.0167). Similarly, for MTHFR (rs1801133) and WNK1 (AluYb8), none of the genotypes were significantly associated with hypertension risk (p > 0.0167).

Overall, after adjustment for multiple comparisons and potential confounders, only BMI and gender emerged as significant independent predictors of hypertension, while the investigated genetic variants did not demonstrate a statistically significant association.

## Discussion

Although the underlying causes of hypertension have not been fully elucidated, several risk factors have been established, including genetic susceptibility, aging and adverse lifestyle choices. An estimation 70-80% of hypertensive cases are associated with unhealthy lifestyles and the presence of multiple risk factors substantially increases the probability of hypertension 29, 30. The development of hypertension involves intricate interactions between environmental exposures and genetic background 31. Numerous polymorphic variants have recently been reported to contribute to susceptibility to hypertension. In this study, we evaluated the potential association of MTHFR (rs1801133, rs1801131) and WNK1 (AluYb8) polymorphisms with hypertension susceptibility in a Jordanian cohort.

The MTHFR enzyme is a critical enzyme involved in homocysteine (Hcy) metabolism. Elevated homocysteine concentrations (hyperhomocysteinemia) are a recognized risk factor for hypertension and cardiovascular disease potentially mediated through vascular endothelial and smooth muscle cell dysfunction 32, 33. The enzymatic activity of MTHFR *in vivo* is strongly influenced by genetic polymorphisms. The MTHFR rs1801133 polymorphism reduces enzyme activity and substantially modifies concentrations of several physiological metabolites, including homocysteine, folic acid and vitamins 34. The folate metabolic pathway is affected by the rs1801133 polymorphism, which decreases enzyme activity and thermostability, resulting in impaired methylation processes 35. Disruption of folate metabolism is linked to elevated plasma homocysteine (Hcy) levels or hyperhomocysteinemia 36, 37. Hyperhomocysteinemia promotes oxidative stress and impairs vascular wall elasticity, leading to endothelial dysfunction, hypertension and related complications 14, 38.

The MTHFR rs1801133 (C677T) polymorphism has been widely investigated in relation to cardiovascular diseases due to its role in folate metabolism and homocysteine regulation. A large meta-analysis demonstrated a significant association between the rs1801133 T allele and increased risk of ischemic stroke across multiple populations, particularly among Asian and middle-aged groups 39. Similarly, this variant has been linked to coronary artery disease, where the T allele was associated with elevated homocysteine levels, increased cardiometabolic risk factors and greater severity of coronary lesions 40. In addition, carriers of this variant have been shown to exhibit unfavorable lipid profiles and inflammatory markers, further supporting its role in cardiovascular risk pathways 40.

Several studies have explored the association between the rs1801133 polymorphism and susceptibility to hypertension. Multiple meta-analyses including ethnically diverse populations have reported that the TT genotype and T allele of rs1801133 are significantly associated with hypertension susceptibility 41-43. Additionally, one meta-analysis demonstrated a positive correlation between this SNP and hypertension in Caucasians and East Asians 44.

The T allele has been associated with an elevated hypertension risk in several populations, including Taiwanese 45, Chinese 46-48, male Spaniards 49, Indians 50, Argentineans 51, 52 and Australian Caucasians 53. Whereas the TT genotype was linked to hypertension in Moroccans 54 and Turkish individuals 55. The C/T genotype appears to be a risk factor in Caucasians 53. Nonetheless, no significant relationship between the rs1801133 and hypertension was reported in Sri Lankan 44, Algerian 56, Caucasian 57, South African 58, Danish 59, Chinese 60, 61, Japanese 62, Latino 44, Black African 44, and Indian 44 populations. Our findings are consistent with those reported in several populations, including Sri Lankan and Danish cohorts; however, they contrast with studies in other ethnic groups, suggesting that genetic background and study design may influence the observed associations.

Another polymorphism of the MTHFR gene is rs1801131, which has been reported in several studies to disrupt homocysteine metabolism and contribute to elevated plasma homocysteine levels 63-65. However, meta-analyses have consistently reported no significant association between the MTHFR rs1801131 polymorphism and hypertension. In a meta-analysis including 1,009 cases and 994 controls, rs1801131 showed no significant relationship with hypertension 42. Similarly, another meta-analysis comprising 11 studies with 2,504 cases and 2,979 controls demonstrated that rs1801131 was not significantly associated with hypertension in either overall or subgroup analyses across all genetic models 44.

Several studies have reported no significant association between the MTHFR rs1801131 polymorphism and hypertension. For instance, no relationship was found between this variant and the development of essential hypertension in Turkish populations 55, and similar negative findings were observed in Northeast Chinese populations 61. In contrast, Markan et al. reported an association between rs1801131 alleles and increased hypertension risk in the Indian population 50. Similarly, the CC genotype and C allele were found to be significantly associated with hypertension in Caucasian subjects 57. In the Qassim region of Saudi Arabia, a higher frequency of the mutant C allele was observed in hypertensive cases compared with controls 66. Moreover, in the Bai population from Yunnan, China, the CC genotype of rs1801131 was shown to significantly elevate hypertension risk 67. Our findings are consistent with studies in Indian, Caucasian and Saudi populations; however, they differ from studies reporting no association, which may be attributed to genetic heterogeneity as well as differences in population characteristics and environmental exposures.

This inconsistency between studies may reflect population-specific genetic architecture, as well as differences in environmental exposures such as dietary folate intake, which may modulate the functional impact of the rs1801131 variant. Furthermore, variations in allele frequencies and linkage disequilibrium patterns among different ethnic groups may contribute to the inconsistent associations observed, highlighting the importance of population-specific investigations. In addition, variations in the linkage disequilibrium between populations around the rs1801131 locus may influence the observed associations and should be considered when interpreting cross-population comparisons.

WNK1 belongs to the serine/threonine kinase family and exerts regulatory effects on several ion channels that contribute to sodium and chloride transport in the kidney 68, 69. The sodium-chloride cotransporter (NCC), located in the distal convoluted tubule (DCT), plays a pivotal role in sodium reabsorption and, when hyperactivated, contributes to volume expansion and the development of hypertension 70, 71. Activation of the sodium-chloride cotransporter (NCC) requires direct phosphorylation by Ste20-like proline-alanine-rich kinase (SPAK) and oxidative stress-responsive kinase 1 (OSR1), both of which are downstream targets activated via phosphorylation by members of the WNK kinase family 72. Mutations in WNK1 have been reported to be associated with upregulated expression of L-WNK1 in the distal tubule, which leads to overactivation of the WNK-SPAK/OSR1-NCC signaling pathway, enhanced NaCl reabsorption, and contributes to hypertension 73.

Several WNK1 polymorphisms have been implicated in blood pressure regulation and hypertension severity 74-76. Among these, the AluYb8 insertion in intron 10 of WNK1 has been relatively understudied with respect to hypertension. Its inclusion in the present study was motivated by previous evidence suggesting its contribution to interindividual variability in blood pressure, particularly in European populations 26, although findings across studies remain inconsistent. However, no association was observed in a Russian Caucasian cohort 77, indicating potential population-specific effects. Thus, evaluating this polymorphism in Jordanians aimed at elucidating the possible involvement of this polymorphism in an unstudied population and gaining insight into genetic differences between populations. Consistent with previous reports, we found no significant link between the AluYb8 insertion polymorphism and hypertension. This lack of association may indicate that the effect of this polymorphism is population-specific or that its contribution to hypertension risk is modest and influenced by interactions with other genetic or environmental factors.

From a clinical perspective, the identification of the MTHFR rs1801131 polymorphism as a potential risk factor may contribute to improved risk stratification for hypertension, particularly in populations with similar genetic backgrounds. However, its clinical applicability remains limited and requires validation in larger, well-characterized cohorts. With regard to the relatively modest effect size and inconsistency in the findings regarding the association between the SNP and hypertension across different populations, rs1801131 alone is not sufficient as a clinical biomarker for predicting the risk of hypertension, and instead, it should be used within a multifactorial risk model. Although MTHFR and WNK1 are involved in distinct biological pathways, potential gene-gene interactions may contribute to hypertension susceptibility and warrant further investigation. To the best of our knowledge, this study represents one of the first investigations evaluating these polymorphisms in a Jordanian population, thereby providing valuable insight into population-specific genetic risk factors.

Gender differences in hypertension were observed in the present study as a secondary finding. In the multivariable regression analysis, Gender was included as a covariate and showed a significant association with hypertension, with higher odds observed in males compared with females. This indicates that Gender may act as an important demographic factor associated with hypertension risk in the studied population. Epidemiological evidence from previous studies has consistently reported age-dependent differences in hypertension prevalence between males and females, with higher risk in males during early and middle adulthood and increasing risk in females after menopause 78. These patterns are thought to be influenced by hormonal, metabolic, and lifestyle-related factors 79. The present findings are consistent with this general epidemiological trend; however, Gender differences were not the primary objective of this study, and no stratified or interaction analyses were performed. Therefore, the observed association should be interpreted as a covariate effect rather than evidence of gender-specific genetic susceptibility.

Beyond that, we identified several limitations despite providing new evidence. Due to the case-control design, the ability to infer causality between the MTHFR rs1801131 polymorphism and hypertension was restricted, and the analysis did not account for all possible environmental and lifestyle factors that may interact with genetic predispositions. More importantly, the study population was relatively homogeneous, which may limit the generalizability of the findings to other ethnic or geographic groups. Despite using regression analysis to adjust for important confounding factors such as age, gender, BMI, and smoking status, the possibility of residual confounding by unmeasured factors cannot be ruled out. Additionally, while Bonferroni correction was applied to address multiple testing, its conservative nature may increase the risk of type II error and may lead to masking true associations. Moreover, the absence of longitudinal clinical data in the form of pre- and post-treatment measures prevented us from assessing the influence of these genetic markers on treatment response. In addition, the availability of clinical parameters was restricted, particularly among control participants, which may have constrained the assessment of genotype-phenotype relationships. Furthermore, plasma homocysteine levels were not measured in this study. Given that the rs1801131 polymorphism is functionally linked to elevated homocysteine concentrations, the absence of these data precluded assessment of the relationship between the genetic variant and hyperhomocysteinemia, a key intermediate phenotype implicated in the pathogenesis of hypertension. Hence, the biology behind how this variant predisposes an individual to the disease was not established.

We suggest that future research should focus on longitudinal study designs and include larger, more diverse populations to validate these findings. Additionally, incorporating biochemical measurements such as homocysteine levels, alongside functional studies, would better understand the molecular mechanisms through which rs1801131 influences blood pressure regulation and may help guide the development of targeted interventions.

## Conclusion

Our study provides preliminary evidence that the MTHFR rs1801131 variant may be associated with an increased risk of hypertension in the studied population. These findings suggest a potential role for this variant in blood pressure regulation, highlighting the importance of considering genetic variability across populations. However, further studies involving larger and more diverse cohorts, as well as functional analyses, are needed to validate these results and clarify the underlying biological mechanisms.

## Figures and Tables

**Table 1:** The primer structure and PCR Product Characteristics.

Polymorphism	Primers	Restriction enzyme	Allele	Fragment size (bp)
MTHFR (rs1801133)	F: 5′-TGAAGGAGAAGGTGTCTGCGGGA-3′ R: 5′-AGGACGGTGCGGTGAGAGTG-3′	HinfI	C T	198 175, 23
MTHFR (rs1801131)	F: 5'-CTTTGGGGAGCTGAAGGACTACTAC-3′ R: 5'-CACTTTGTGACCATTCCGGTTTG-3′	MboII	A C	56, 31, 30, 28, 18 84, 31, 30, 18
WNK1 (AluYb8)	F: 5′-GGGTAACCAACCCTTGAAGTAGG-3′ R: 5′-GGGTACTTCTCAAGTGATTAGGAGGA-3′	_	I D	640 353

**Table 2:** Comparison of baseline characteristics between hypertensive and control groups.

Characteristics		Male control (n=82)	Male case (n=128)	P-value	Female control (n=138)	Female case (n=92)	P-value
Age (Mean ± SD)		58.70 ± 10.27	57.95 ± 10.12	0.601	58.78 ± 10.32	60.29 ± 10.28	0.290
Body Mass Index (kg/m²)	(Mean ± SD)	26.37 ± 4.26	29.85 ± 4.78	< 0.0001*	24.25 ± 4.10	34.02 ± 6.75	< 0.0001*
	Underweight	2 (2.4%)	0 (0%)	0.0009*	7 (5.1%)	0 (0%)	< 0.0001*
	Normal weight	33 (40.3%)	24 (18.8%)		79 (57.2%)	7 (7.6%)	
	Overweight	32 (39%)	39 (30.4%)		37 (26.8%)	19 (20.7%)	
	Obesity	15 (18.3%)	65 (50.8%)		15 (10.9%)	66 (71.7%)	
Smoker	Yes	41 (50%)	52 (40.6%)	0.202	5 (3.6%)	6 (6.5%)	0.355
	No	41 (50%)	76 (59.4%)		133 (96.4%)	86 (93.5%)	

**Table 3:** Minor allele frequencies and Hardy-Weinberg equilibrium analysis in cases and controls.

Polymorphism	Control (n=220)			Cases (n=220)		
	MA	MAF	HWE p-value	MA	MAF	HWE p-value
MTHFR (rs1801133)	T	34%	0.55	T	35%	0.18
MTHFR (rs1801131)	C	20%	1	C	32%	0.44
WNK1 (AluYb8)	I	15%	0.12	I	11%	0.32

^MA^ Minor Allele^MAF^ Minor Allele Frequency

**Table 4:** Genotype distribution and relative allele frequency for MTHFR and WNK1 polymorphisms.

Polymorphism	Allele/ Genotype	Cases (N = 220)	Control (N = 220)	p-value*	Chi-square (χ²)
MTHFR (rs1801133)	T C	154 (35%) 286 (65%)	150 (34%) 290 (66%)	0.77	0.08
	TT CT CC	22 (10%) 110 (50%) 88 (40%)	23 (10%) 104 (47%) 93 (42%)	0.84	0.32
MTHFR (rs1801131)	C A	140 (32%) 300 (68%)	89 (20%) 351 (80%)	0.0001*	15.35
	CC AC AA	25 (11%) 90 (41%) 105 (48%)	9 (4%) 71 (32%) 140 (64%)	0.0006*	14.77
WNK1 (AluYb8)	I D	50 (11%) 390 (89%)	68 (15%) 372 (85%)	0.07	3.17
	II ID DD	1 (0%) 48 (22%) 171 (78%)	2 (1%) 64 (29%) 154 (70%)	0.17	3.50

p-values were calculated using the chi-square (χ²) test for comparison of allele and genotype frequencies between groups. * P-values < 0.0167 (0.05/# of SNPs, 0.05/3 = 0.0167 after applying multiple comparisons) are considered significant.

**Table 5:** Genetic model analyses of MTHFR and WNK1 polymorphisms in cases and controls.

Polymorphism	Model	Genotype	Cases (%)	Controls (%)	OR (95% CI)	p- value
*MTHFR* (rs1801133)	Codominant	C/C C/T T/T	88 (40%) 110 (50%) 22 (10%)	93 (42.3%) 104 (47.3%) 23 (10.4%)	1.00 1.12 (0.75-1.66) 1.01 (0.53-1.94)	0.85
	Dominant	C/C C/T-T/T	88 (40%) 132 (60%)	93 (42.3%) 127 (57.7%)	1.00 1.10 (0.75-1.61)	0.63
	Recessive	C/C-C/T T/T	198 (90%) 22 (10%)	197 (89.5%) 23 (10.4%)	1.00 0.95 (0.51-1.76)	0.88
	Overdominant	C/C/T/T C/T	110 (50%) 110 (50%)	116 (52.7%) 104 (47.3%)	1.00 1.12 (0.77-1.62)	0.57
MTHFR (rs1801131)	Codominant	A/A A/C C/C	105 (47.7%) 90 (40.9%) 25 (11.4%)	140 (63.6%) 71 (32.3%) 9 (4.1%)	1.00 1.69 (1.13-2.52) 3.70 (1.66-8.27)	5e-04*
	Dominant	A/A A/C-C/C	105 (47.7%) 115 (52.3%)	140 (63.6%) 80 (36.4%)	1.00 1.92 (1.31-2.81)	8e-04*
	Recessive	A/A-A/C C/C	195 (88.6%) 25 (11.4%)	211 (95.9%) 9 (4.1%)	1.00 3.01 (1.37-6.60)	0.0036*
	Overdominant	A/A-C/C A/C	130 (59.1%) 90 (40.9%)	149 (67.7%) 71 (32.3%)	1.00 1.45 (0.98-2.15)	0.06
WNK1 (AluYb8)	Codominant	D/D I/D I/I	171 (77.7%) 48 (21.8%) 1 (0.4%)	154 (70%) 64 (29.1%) 2 (0.9%)	1.00 0.68 (0.44-1.04) 0.45 (0.04-5.02)	0.17
	Dominant	D/D I/D-I/I	171 (77.7%) 49 (22.3%)	154 (70%) 66 (30%)	1.00 0.67 (0.44-1.03)	0.065
	Recessive	D/D-I/D I/I	219 (99.5%) 1 (0.4%)	218 (99.1%) 2 (0.9%)	1.00 0.50 (0.04-5.52)	0.56
	Overdominant	D/D-I/I I/D	172 (78.2%) 48 (21.8%)	156 (70.9%) 64 (29.1%)	1.00 0.68 (0.44-1.05)	0.08

* P-values < 0.0167 (0.05/# of SNPs, 0.05/3 = 0.0167 after applying multiple comparisons) are considered significant.

**Table 6:** Association between hypertension and different MTHFR haplotypes.

Haplotype	Total	Control	Cases	OR (95 % CI)	p-value
C A	0.4059	0.4695	0.3433	1.00	---
T A	0.3339	0.3282	0.3385	1.29 (0.94 - 1.77)	0.12
C C	0.2486	0.1896	0.3067	2.02 (1.43 - 2.85)	1e-04*
T C	0.0116	0.0127	0.0115	1.26 (0.21 - 7.46)	0.8

* P-values < 0.0167 (0.05/# of SNPs, 0.05/3 = 0.0167 after applying multiple comparisons) are considered significant.

**Table 7:** Association between studied polymorphism genotypes and clinical outcomes in the hypertension patient group.

Clinical Outcome	WNK1 (AluYb8) II/ID/DD	MTHFR (rs1801133) TT/TC/CC	MTHFR (rs1801131) AA/AC/CC
Age	0.260^a^ 1.353^b^	0.157^a^ 1.868^b^	0.477^a^ 0.743^b^
Gender	0.421^a^ 1.728^c^	0.901^a^ 0.206^c^	0.476^a^ 1.481^c^
BMI	0.332^a^ 1.452^b^	0.132^a^ 1.964^b^	0.611^a^ 0.577^b^
Newly Diagnosed HTN	0.522^a^ 1.299^c^	0.874^a^ 0.267^c^	0.278^a^ 2.557^c^
Know HTN	0.522^a^ 1.299^c^	0.874^a^ 0.267^c^	0.278^a^ 2.557c
Number of Years of HTN	0.700^a^ 0.357^b^	0.633^a^ 0.457^b^	0.924^a^ 0.078^b^
Systolic Blood Pressure (SBP)	0.963^a^ 0.037^b^	0.746^a^ 0.292^b^	0.138^a^ 1.995^b^
Diastolic Blood Pressure (DBP)	0.073^a^ 2.640^b^	0.497^a^ 0.701^b^	0.248^a^ 1.403^b^
Pulse Rate	0.014^a^ 4.314^b^	0.994^a^ 0.005^b^	0.672^a^ 0.397^b^
Diabetes Mellitus	0.667^a^ 0.807^c^	0.293^a^ 2.449^c^	0.125^a^ 4.154^c^
Number of Years DM	0.559^a^ 0.583^b^	0.257^a^ 1.366^b^	0.542^a^ 0.612^b^
Diabetes Mellitus Treatment	0.542^a^ 1.225^c^	0.243^a^ 2.823^c^	0.106^a^ 4.478^c^
Ischemic Heart Disease	0.2911^a^ 2.468^c^	0.4339^a^ 1.670^c^	0.6234^a^ 0.9450^c^
Heart Failure	0.851^a^ 0.320^c^	0.148^a^ 3.812^c^	0.223^a^ 2.054^c^
Peripheral Vascular Disease	0.804^a^ 0.434^c^	0.278^a^ 2.558^c^	0.801^a^ 0.443^c^
Cerebrovascular Accident	0.701^a^ 0.708^c^	0.936^a^ 0.131^c^	0.431^a^ 1.683^c^
Chronic Kidney Disease	0.266^a^ 2.642^c^	0.933^a^ 0.157^c^	0.087^a^ 4.878^c^
Dialysis	0.636^a^ 0.903^c^	0.372^a^ 1.977^c^	0.556^a^ 1.171^c^
Atrial Fibrillation	0.031^a^ 6.945^c^	0.392^a^ 1.870^c^	0.649^a^ 0.864^c^
Smoker	0.738^a^ 0.605^c^	0.750^a^ 0.574^c^	0.459^a^ 1.554^c^
Ex-smoker	0.561^a^ 1.154^c^	0.897^a^ 0.217^c^	0.333^a^ 2.198^c^
Diet	0.795^a^ 0.458^c^	0.054^a^ 5.811^c^	0.415^a^ 1.755^c^
Exercises	0.852^a^ 0.3181^c^	0.172^a^ 3.521^c^	0.303^a^ 2.386^c^
Hemoglobin (HB)	0.908^a^ 0.096^b^	0.788^a^ 0.238^b^	0.827^a^ 0.189^b^
White Blood Cells (WBCs)	0.171^a^ 1.783^b^	0.659^a^ 0.418^b^	0.733^a^ 0.310^b^
Platelets	0.730^a^ 0.315^b^	0.249^a^ 1.401^b^	0.380^a^ 0.970^b^
Na^+^	0.409^a^ 0.897^b^	0.588^a^ 0.531^b^	0.053^a^ 2.973^b^
K^+^	0.405^a^ 0.907^b^	0.173^a^ 1.768^b^	0.554^a^ 0.592^b^
Urea	0.553^a^ 0.988^b^	0.963^a^ 0.037^b^	0.216^a^ 1.545^b^
Creatinine	0.455^a^ 0.789^b^	0.337^a^ 1.091^b^	0.352^a^ 1.047^b^
Total Cholesterol	0.831^a^ 0.185^b^	0.456^a^ 0.787^b^	0.636^a^ 0.453^b^
LDL	0.226^a^ 1.501^b^	0.743^a^ 0.296^b^	0.853^a^ 0.159^b^
HDL	0.008^a^ 4.996^b^	0.575^a^ 0.554^b^	0.995^a^ 0.004^b^
Triglyceride	0.101^a^ 2.322^b^	0.581^a^ 0.544^b^	0.575^a^ 0.555^b^
Glucose	0.161^a^ 1.850^b^	0.167^a^ 1.815^b^	0.333^a^ 1.108^b^
HBA1C	0.010^a^ 4.722^b^	0.254^a^ 1.381^b^	0.197^a^ 1.642^b^
Thyroid Stimulating Hormone (TSH)	0.089^a^ 2.468^b^	0.569^a^ 0.566^b^	0.695^a^ 0.364^b^
Albumin	0.334^a^ 1.103^b^	0.561^a^ 0.578^b^	0.099^a^ 2.345^b^
Total Protein	0.359^a^ 1.031^b^	0.095^a^ 2.382^b^	0.689^a^ 0.372^b^

^a^ P-values < 0.0167 (0.05/# of SNPs, 0.05/3 = 0.0167 after applying multiple comparisons) are considered significant.^b^ ANOVA-derived F-value.^c^ Pearson chi-squared-derived χ² value.

**Table 8:** Multivariable Binary Logistic Regression Analysis of Genetic Polymorphisms and Covariates Associated with Hypertension Risk.

Polymorphism**	Covariate	Odd ratio	Confidence interval 95 %	P value*
***MTHFR*** **(rs1801131)**	Age	1.007	0.985 - 1.030	0.521
	BMI	1.295	1.228 - 1.365	0.000*
	Gender	3.190	1.898 - 5.365	0.000*
	Smoking	0.481	0.251 - 0.924	0.028
	C/C	2.637	1.032 - 6.737	0.043
	A/C	1.718	1.045 - 2.824	0.033
	A/A	Reference		
***MTHFR*** **(rs1801133)**	Age	1.006	0.984 - 1.029	0.584
	BMI	1.302	1.234 - 1.374	0.000*
	Gender	3.604	2.141 - 6.068	0.000*
	Smoking	0.471	0.248 - 0.894	0.021
	T/T	1.043	0.455 - 2.392	0.921
	C/T	1.499	0.908 - 2.474	0.113
	C/C	Reference		
***WNK1*** **(AluYb8)**	Age	1.006	0.983 - 1.029	0.624
	BMI	1.296	1.230 - 1.366	0.000*
	Gender	3.435	2.054 - 5.745	0.000*
	Smoking	0.462	0.244 - 0.874	0.018
	I/I	1.103	0.041 - 29.321	0.953
	I/D	0.637	0.371 - 1.093	0.102
	D/D	Reference		

**The reference category is the control. * P-values < 0.0167 (0.05/# of SNPs, 0.05/3 = 0.0167 after applying multiple comparisons) are considered significant.

## Data Availability

The data that support the findings of this study are available from the corresponding author upon reasonable request.

## References

[1] Kearney PM, Whelton M, Reynolds K, Muntner P, Whelton PK, He J (2005). Global burden of hypertension: analysis of worldwide data. Lancet.

[2] Padmanabhan S, Paul L, Dominczak AF (2010). The Pharmacogenomics of Anti-Hypertensive Therapy. Pharmaceuticals (Basel).

[3] Chow CK, Teo KK, Rangarajan S, Islam S, Gupta R, Avezum A (2013). Prevalence, awareness, treatment, and control of hypertension in rural and urban communities in high-, middle-, and low-income countries. JAMA.

[4] Oparil S, Zaman MA, Calhoun DA (2003). Pathogenesis of hypertension. Ann Intern Med.

[5] Ibrahim MM, Damasceno A (2012). Hypertension in developing countries. Lancet.

[6] Staessen JA, Wang J, Bianchi G, Birkenhäger WH (2003). Essential hypertension. The Lancet.

[7] Mills KT, Stefanescu A, He J (2020). The global epidemiology of hypertension. Nat Rev Nephrol.

[8] Beevers G, Lip GY, O'Brien E (2001). ABC of hypertension: The pathophysiology of hypertension. BMJ.

[9] Tabara Y, Kohara K, Miki T; Millennium Genome Project for Hypertension (2012). Hunting for genes for hypertension: the Millennium Genome Project for Hypertension. Hypertens Res.

[10] Hu XJ, Su MR, Cao BW, Ou FB, Yin RX, Luo AD (2023). Relationship between the methylenetetrahydrofolate reductase (MTHFR) rs1801133 SNP and serum homocysteine levels of Zhuang hypertensive patients in the central region of Guangxi. Clin Hypertens.

[11] Ornosa-Martín G, Fernandez-Ballart JD, Ceruelo S, Ríos L, Ueland PM, Meyer K (2020). Homocysteine, the methylenetetrahydrofolate reductase 677C>T polymorphism and hypertension: effect modifiers by lifestyle factors and population subgroups. Br J Nutr.

[12] Yuan X, Wang T, Gao J, Wang Y, Chen Y, Kaliannan K (2020). Associations of homocysteine status and homocysteine metabolism enzyme polymorphisms with hypertension and dyslipidemia in a Chinese hypertensive population. Clin Exp Hypertens.

[13] Nam KW, Kwon HM, Jeong HY, Park JH, Kwon H, Jeong SM (2019). Serum homocysteine level is related to cerebral small vessel disease in a healthy population. Neurology.

[14] Fu L, Li YN, Luo D, Deng S, Wu B, Hu YQ (2019). Evidence on the causal link between homocysteine and hypertension from a meta-analysis of 40 173 individuals implementing Mendelian randomization. J Clin Hypertens (Greenwich).

[15] Balint B, Jepchumba VK, Guéant JL, Guéant-Rodriguez RM (2020). Mechanisms of homocysteine-induced damage to the endothelial, medial and adventitial layers of the arterial wall. Biochimie.

[16] Yuyun MF, Ng LL, Ng GA (2018). Endothelial dysfunction, endothelial nitric oxide bioavailability, tetrahydrobiopterin, and 5-methyltetrahydrofolate in cardiovascular disease. Where are we with therapy?. Microvasc Res.

[17] Weisberg IS, Jacques PF, Selhub J, Bostom AG, Chen Z, Curtis Ellison R (2001). The 1298A->C polymorphism in methylenetetrahydrofolate reductase (MTHFR): *in vitro* expression and association with homocysteine. Atherosclerosis.

[18] Rozen R (1997). Genetic predisposition to hyperhomocysteinemia: deficiency of methylenetetrahydrofolate reductase (MTHFR). Thromb Haemost.

[19] Luo Z, Lu Z, Muhammad I, Chen Y, Chen Q, Zhang J, Song Y (2018). Associations of the MTHFR rs1801133 polymorphism with coronary artery disease and lipid levels: a systematic review and updated meta-analysis. Lipids Health Dis.

[20] Fung MM, Salem RM, Lipkowitz MS, Bhatnagar V, Pandey B, Schork NJ (2012). Methylenetetrahydrofolate reductase (MTHFR) polymorphism A1298C (Glu429Ala) predicts decline in renal function over time in the African-American Study of Kidney Disease and Hypertension (AASK) Trial and Veterans Affairs Hypertension Cohort (VAHC). Nephrol Dial Transplant.

[21] Soleimani-Jadidi S, Meibodi B, Javaheri A, Tabatabaei RS, Hadadan A, Zanbagh L (2022). Association between Fetal MTHFR A1298C (rs1801131) Polymorphism and Neural Tube Defects Risk: A Systematic Review and Meta-Analysis. Fetal Pediatr Pathol.

[22] Xu B, English JM, Wilsbacher JL, Stippec S, Goldsmith EJ, Cobb MH (2000). WNK1, a novel mammalian serine/threonine protein kinase lacking the catalytic lysine in subdomain II. J Biol Chem.

[23] Wilson FH, Disse-Nicodème S, Choate KA, Ishikawa K, Nelson-Williams C, Desitter I (2001). Human hypertension caused by mutations in WNK kinases. Science.

[24] Delaloy C, Lu J, Houot AM, Disse-Nicodeme S, Gasc JM, Corvol P (2003). Multiple promoters in the WNK1 gene: one controls expression of a kidney-specific kinase-defective isoform. Mol Cell Biol.

[25] Veríssimo F, Jordan P (2001). WNK kinases, a novel protein kinase subfamily in multi-cellular organisms. Oncogene.

[26] Putku M, Kepp K, Org E, Sõber S, Comas D, Viigimaa M (2011). Novel polymorphic AluYb8 insertion in the WNK1 gene is associated with blood pressure variation in Europeans. Hum Mutat.

[27] Nyholt DR (2004). A simple correction for multiple testing for single-nucleotide polymorphisms in linkage disequilibrium with each other. Am J Hum Genet.

[28] Li J, Ji L (2005). Adjusting multiple testing in multilocus analyses using the eigenvalues of a correlation matrix. Heredity.

[29] Carlene MM, Lawes SVH, Rodgers A (2008). Global burden of bloodpressure-related disease. Lancet.

[30] Tanira MO, Al Balushi KA (2005). Genetic variations related to hypertension: a review. J Hum Hypertens.

[31] Waken RJ, de las Fuentes L, Rao DC (2017). A Review of the Genetics of Hypertension with a Focus on Gene-Environment Interactions. Curr Hypertens Rep.

[32] Jakubowski H (2008). The pathophysiological hypothesis of homocysteine thiolactone-mediated vascular disease. J Physiol Pharmacol.

[33] Ganguly P, Alam SF (2015). Role of homocysteine in the development of cardiovascular disease. Nutr J.

[34] Zaric BL, Obradovic M, Bajic V, Haidara MA, Jovanovic M, Isenovic ER (2019). Homocysteine and Hyperhomocysteinaemia. Curr Med Chem.

[35] Frosst P, Blom HJ, Milos R, Goyette P, Sheppard CA, Matthews RG (1995). A candidate genetic risk factor for vascular disease: a common mutation in methylenetetrahydrofolate reductase. Nat Genet.

[36] Brattström L, Wilcken DE, Ohrvik J, Brudin L (1998). Common methylenetetrahydrofolate reductase gene mutation leads to hyperhomocysteinemia but not to vascular disease: the result of a meta-analysis. Circulation.

[37] Pereira AC, Schettert IT, Morandini Filho AA, Guerra-Shinohara EM, Krieger JE (2004). Methylenetetrahydrofolate reductase (MTHFR) c677t gene variant modulates the homocysteine folate correlation in a mild folate-deficient population. Clin Chim Acta.

[38] Rodrigo R, González J, Paoletto F (2011). The role of oxidative stress in the pathophysiology of hypertension. Hypertens Res.

[39] Zhao L, Li T, Dang M, Li Y, Fan H, Hao Q (2023). Association of methylenetetrahydrofolate reductase (MTHFR) rs1801133 (677C>T) gene polymorphism with ischemic stroke risk in different populations: An updated meta-analysis. Front Genet.

[40] Luo Z, Tang K, Huang G, Wang X, Zhou S, Dai D (2024). Homocysteine concentration in coronary artery disease and severity of coronary lesions. J Cell Mol Med.

[41] Fan Y, Wu L, Zhuang W (2022). Methylenetetrahydrofolate Reductase Gene rs1801133 and rs1801131 Polymorphisms and Essential Hypertension Risk: A Comprehensive Analysis. Cardiovasc Ther.

[42] Wu YL, Hu CY, Lu SS, Gong FF, Feng F, Qian ZZ (2014). Association between methylenetetrahydrofolate reductase (MTHFR) C677T/A1298C polymorphisms and essential hypertension: a systematic review and meta-analysis. Metabolism.

[43] Yang KM, Jia J, Mao LN, Men C, Tang KT, Li YY (2014). Methylenetetrahydrofolate reductase C677T gene polymorphism and essential hypertension: A meta-analysis of 10,415 subjects. Biomed Rep.

[44] Yang B, Fan S, Zhi X, Li Y, Liu Y, Wang D (2014). Associations of MTHFR gene polymorphisms with hypertension and hypertension in pregnancy: a meta-analysis from 114 studies with 15411 cases and 21970 controls. PLoS One.

[45] Lin PT, Cheng CH, Wei JC, Huang YC (2008). Low plasma pyridoxal 5'-phosphate concentration and MTHFR 677C->T genotypes are associated with increased risk of hypertension. Int J Vitam Nutr Res.

[46] Wen C, Lv JF, Wang L, Zhu WF, Wan FS, Wang XZ (2015). Association of a methylene tetrahydrofolate reductase C677T polymorphism with several blood chemical levels in a Chinese population. Genet Test Mol Biomarkers.

[47] Wu H, Huang Q, Yu Z, Zhong Z (2022). Association of ALDH2 rs671 and MTHFR rs1801133 polymorphisms with hypertension among Hakka people in Southern China. BMC Cardiovasc Disord.

[48] Cai W, Yin L, Yang F, Zhang L, Cheng J (2014). Association between Hcy levels and the CBS844ins68 and MTHFR C677T polymorphisms with essential hypertension. Biomed Rep.

[49] Rodríguez-Esparragón F, Hernández-Perera O, Rodríguez-Pérez JC, Anábitarte A, Díaz-Cremades JM, Losada A (2003). The effect of methylenetetrahydrofolate reductase C677T common variant on hypertensive risk is not solely explained by increased plasma homocysteine values. Clin Exp Hypertens.

[50] Markan S, Sachdeva M, Sehrawat BS, Kumari S, Jain S, Khullar M (2007). MTHFR 677 CT/MTHFR 1298 CC genotypes are associated with increased risk of hypertension in Indians. Mol Cell Biochem.

[51] Fridman O, Porcile R, Vanasco V, Junco MN, Gariglio L, Potenzoni MA (2008). Study on homocysteine levels and methylenetetrahydrofolate reductase gene variant (C677T) in a population of Buenos Aires City. Clin Exp Hypertens.

[52] Fridman O, Porcile R, Morales AV, Gariglio LO, Potenzoni MA, Turk Noceto PC (2013). Association of methylenetetrahydrofolate reductase gene 677C>T polymorphism with hypertension in older women in a population of Buenos Aires City. Clin Exp Hypertens.

[53] Heux S, Morin F, Lea RA, Ovcaric M, Tajouri L, Griffiths LR (2004). The methylentetrahydrofolate reductase gene variant (C677T) as a risk factor for essential hypertension in Caucasians. Hypertens Res.

[54] Nassereddine S, Kassogue Y, Korchi F, Habbal R, Nadifi S (2015). Association of methylenetetrahydrofolate reductase gene (C677T) with the risk of hypertension in Morocco. BMC Res Notes.

[55] Er ZC, Muderrisoglu A, Ekim M, Ekim H (2022). MTHFR C677T (rs1801133) genetic polymorphism is associated with the development risk of essential hypertension in the Turkish population. Egypt J Med Hum Genet.

[56] Amrani-Midoun A, Kiando SR, Treard C, Jeunemaitre X, Bouatia-Naji N (2016). The relationship between MTHFR C677T gene polymorphism and essential hypertension in a sample of an Algerian population of Oran city. Int J Cardiol.

[57] Fowdar JY, Lason MV, Szvetko AL, Lea RA, Griffiths LR (2012). Investigation of homocysteine-pathway-related variants in essential hypertension. Int J Hypertens.

[58] Mabhida SE, Sharma JR, Apalata T, Masilela C, Nomatshila S, Mabasa L (2022). The association of MTHFR (rs1801133) with hypertension in an indigenous south African population. Front Genet.

[59] Husemoen LL, Skaaby T, Jørgensen T, Thuesen BH, Fenger M, Grarup N (2014). MTHFR C677T genotype and cardiovascular risk in a general population without mandatory folic acid fortification. Eur J Nutr.

[60] Zhan S, Gao Y, Yin X, Huang Y, Hu Y, Li L (2000). [A case-control study on the relationship between abnormal homocysteine metabolism and essential hypertension]. Zhonghua Liu Xing Bing Xue Za Zhi.

[61] Liu S, Liu M, Li Q, Liu X, Wang Y, Mambiya M (2019). Association of single nucleotide polymorphisms of MTHFR, TCN2, RNF213 with susceptibility to hypertension and blood pressure. Biosci Rep.

[62] Lwin H, Yokoyama T, Yoshiike N, Saito K, Yamamoto A, Date C (2006). Polymorphism of methylenetetrahydrofolate reductase gene (C677T MTHFR) is not a confounding factor of the relationship between serum uric acid level and the prevalence of hypertension in Japanese men. Circ J.

[63] Fekih-Mrissa N, Mrad M, Ibrahim H, Akremi I, Sayeh A, Jaidane A (2017). Methylenetetrahydrofolate Reductase (MTHFR) (C677T and A1298C) Polymorphisms and Vascular Complications in Patients with Type 2 Diabetes. Can J Diabetes.

[64] Jadeja SD, Mansuri MS, Singh M, Patel H, Marfatia YS, Begum R (2018). Association of elevated homocysteine levels and Methylenetetrahydrofolate reductase (MTHFR) 1298 A > C polymorphism with Vitiligo susceptibility in Gujarat. J Dermatol Sci.

[65] Böttiger AK, Hurtig-Wennlöf A, Sjöström M, Yngve A, Nilsson TK (2007). Association of total plasma homocysteine with methylenetetrahydrofolate reductase genotypes 677C>T, 1298A>C, and 1793G>A and the corresponding haplotypes in Swedish children and adolescents. Int J Mol Med.

[66] Alghasham A, Settin AA, Ali A, Dowaidar M, Ismail H (2012). Association of MTHFR C677T and A1298C gene polymorphisms with hypertension. Int J Health Sci (Qassim).

[67] Liu Y, Xu C, Wang Y, Yang C, Pu G, Zhang L (2023). Association analysis of MTHFR (rs1801133 and rs1801131) and MTRR (rs1801394) gene polymorphisms towards the development of hypertension in the Bai population from Yunnan, China. Clin Exp Hypertens.

[68] Náray-Fejes-Tóth A, Snyder PM, Fejes-Tóth G (2004). The kidney-specific WNK1 isoform is induced by aldosterone and stimulates epithelial sodium channel-mediated Na+ transport. Proc Natl Acad Sci U S A.

[69] Cope G, Murthy M, Golbang AP, Hamad A, Liu CH, Cuthbert AW (2006). WNK1 affects surface expression of the ROMK potassium channel independent of WNK4. J Am Soc Nephrol.

[70] Simon DB, Nelson-Williams C, Bia MJ, Ellison D, Karet FE, Molina AM (1996). Gitelman's variant of Bartter's syndrome, inherited hypokalaemic alkalosis, is caused by mutations in the thiazide-sensitive Na-Cl cotransporter. Nat Genet.

[71] Subramanya AR, Ellison DH (2014). Distal convoluted tubule. Clin J Am Soc Nephrol.

[72] Richardson C, Rafiqi FH, Karlsson HK, Moleleki N, Vandewalle A, Campbell DG, Morrice NA, Alessi DR (2008). Activation of the thiazide-sensitive Na+-Cl- cotransporter by the WNK-regulated kinases SPAK and OSR1. J Cell Sci.

[73] Brown A, Meor Azlan NF, Wu Z, Zhang J (2021). WNK-SPAK/OSR1-NCC kinase signaling pathway as a novel target for the treatment of salt-sensitive hypertension. Acta Pharmacol Sin.

[74] Han YF, Hui RT (2008). The role of WNK1 gene Pro1056Thr polymorphism in essential hypertension. Mol. Cardiol. China.

[75] Kokubo Y, Kamide K, Inamoto N, Tanaka C, Banno M, Takiuchi S (2004). Identification of 108 SNPs in TSC, WNK1, and WNK4 and their association with hypertension in a Japanese general population. J Hum Genet.

[76] Tobin MD, Raleigh SM, Newhouse S, Braund P, Bodycote C, Ogleby J (2005). Association of WNK1 gene polymorphisms and haplotypes with ambulatory blood pressure in the general population. Circulation.

[77] Akhmedova E, Nikulina SY, Salmina AB, Chernova A, Bazanova M, Ohapkina A (2017). AluYb8 insertion in the WNK1 gene is not associated with hypertension in a Russian caucasian population. Genet. Mol. Res.

[78] Mousavi SS, Guo Y, Robichaux C, Sarker A, Sameni R (2024). Learning from Two Decades of Blood Pressure Data: Demography-Specific Patterns Across 75 Million Patient Encounters. Annu Int Conf IEEE Eng Med Biol Soc.

[79] Vriend EMC, Galenkamp H, van Valkengoed IGM, van den Born BH (2024). Sex disparities in hypertension prevalence, blood pressure trajectories and the effects of anti-hypertensive treatment. Blood Press.

